# Relative effects of interval training with different intensities and interval structures on cardiometabolic health indicators in sedentary young males: a randomized controlled trial

**DOI:** 10.3389/fphys.2026.1842740

**Published:** 2026-06-04

**Authors:** Xudong Shen, Siyu Wang, Rite Ge, Ming Wei

**Affiliations:** School of Strength and Conditioning, Shenyang Sport University, Shenyang, China

**Keywords:** body composition, cardiometabolic health, interval training, lipid profile, sedentary population

## Abstract

**Objective:**

Sedentary behavior is closely associated with early cardiometabolic dysfunction in young populations. However, under approximately matched prescribed active-work-load conditions, it remains unclear whether interval-training protocols differing in exercise intensity and interval structure induce different cardiometabolic responses in sedentary individuals.

**Methods:**

Forty sedentary young males (22.85 ± 0.22 years) were randomized to four 8-week interval-training protocols: moderate-intensity short-interval group (MISI: 60%–70% maximal heart rate (HRmax), 60 s work/30 s rest), moderate-intensity long-interval group (MILI: 60%–70% HRmax, 60 s work/120 s rest), high-intensity short-interval group (HISI: 85%–95% HRmax, 30 s work/30 s rest), and high-intensity long-interval group (HILI: 85%–95% HRmax, 30 s work/120 s rest) (n = 10 per group). Two-way repeated-measures ANOVA was used to compare pre–post changes in body fat percentage (BFP), skeletal muscle mass (SMM), visceral fat area (VFA), basal metabolic rate (BMR), total cholesterol (TC), triglycerides (TG), low-density lipoprotein cholesterol (LDL-C), and high-density lipoprotein cholesterol (HDL-C).

**Results:**

BFP, VFA, TC, and TG decreased significantly in all four protocols, whereas SMM and HDL-C increased significantly. BMR increased significantly in MILI, HISI, and HILI, but not in MISI. VFA decreased in all groups, and post-intervention VFA was significantly lower in HISI than in MILI (*p* < 0.05). TG decreased in all groups, and post-intervention TG was significantly lower in HISI than in MISI (*p* < 0.05). Improvements in LDL-C were observed only in the high-intensity training groups (*p* < 0.05). HDL-C increased significantly in all four protocols, and post-intervention HDL-C was significantly higher in HISI and HILI than in MILI.

**Conclusion:**

Eight weeks of interval training was associated with favorable changes in selected body composition and lipid-profile indicators in sedentary young males. Compared with the moderate-intensity protocols, the high-intensity protocols showed more favorable changes in selected outcomes, including VFA, TG, and LDL-C. The HISI protocol may represent a promising option for improving selected cardiometabolic risk indicators related to visceral adiposity and lipid metabolism in sedentary young males; however, these findings should be interpreted cautiously given the small sample size, the absence of a non-exercise control group, and the lack of direct physiological-load measurements.

## 1 Introduction

Sedentary behavior is defined as any waking behavior characterized by sitting, reclining, or lying postures with an energy expenditure of ≤ 1.5 metabolic equivalents (Tremblay et al., 2017a). With the transformation of modern lifestyles, sedentary behavior has become a global phenomenon (Bull et al., 2020). Studies indicate that individuals spend 55%–60% of their waking hours in a sedentary state (Tremblay et al., 2017b; Shibata et al., 2026). The World Health Organization has noted that prolonged sitting may significantly increase the risk of all-cause mortality, cardiovascular mortality, and cancer-related mortality, and is closely associated with the incidence of cardiovascular disease, type 2 diabetes, and cancer (Bull et al., 2020; Higgins et al., 2022). Accordingly, sedentary behavior has been recognized as an important independent health risk factor, distinct from physical activity level (Patterson et al., 2018).

Prolonged sitting may impair lower-limb muscle pump function, reduces blood flow shear stress, disrupt vascular endothelial homeostasis and nitric oxide (NO) bioavailability, and induce an oxidative stress environment, ultimately leading to a decline in endothelium-dependent vasodilation (Thosar et al., 2012). Simultaneously, sedentary behavior interferes with energy and lipid metabolism regulation, inducing central adiposity and dyslipidemia, thereby further exacerbating cardiovascular risk (Onagbiye et al., 2024). Therefore, developing efficient and feasible exercise intervention strategies for sedentary populations is of major public health significance.

Current interventions targeting sedentary populations primarily fall into two categories: reducing sedentary time, and improving metabolic risk through regular exercise. However, in real-world settings, sedentary individuals often struggle to sustain continuous aerobic training over extended periods due to time constraints, insufficient exercise experience, and poor adherence. By contrast, interval training—which alternates between high-load bouts and recovery phases—has been reported to improve cardiorespiratory fitness, enhance vascular endothelial function, and reduce arterial stiffness and resting blood pressure within a relatively short time (Ramos et al., 2015; Macinnis and Gibala, 2017), making it a compelling option for this population.

The effects of interval training are influenced by several prescription variables, particularly exercise intensity and interval structure (Wen et al., 2019). High-intensity interval training (HIIT) has been reported to produce greater improvements in cardiorespiratory fitness and some vascular outcomes than moderate-intensity continuous training (MICT) in certain populations, although it also imposes greater physiological demands (Milanović et al., 2015; Ramos et al., 2015). In contrast, moderate-intensity interval training may be more tolerable and may support adherence in individuals with limited exercise experience (Masuki et al., 2015; Coswig et al., 2020). Regarding interval structure, shorter work–rest cycles appear to favor maximal oxygen uptake and vascular adaptation (Buchheit and Laursen, 2013a; Buchheit and Laursen, 2013b), whereas longer cycles may facilitate stable induction of peak output and vascular remodeling signals (Rønnestad et al., 2015). The work-to-rest ratio may shape the cumulative metabolic stress profile of a session (Fuertes-Kenneally et al., 2023).

Although previous studies have examined the effects of exercise intensity or interval structure separately, direct comparisons of combined intensity–interval configurations remain limited in sedentary young males. Therefore, this study aimed to compare the relative effects of four active interval-training protocols combining moderate or high intensity with short or long interval structures on body composition and lipid profile indicators in this population.

## 2 Participants and methods

### 2.1 Participants

The required sample size was calculated using G*Power 3.1 (Guo et al., 2013). The statistical test was set as ANOVA: repeated measures, within–between interaction, corresponding to the primary group × time analytical framework of the present four-arm pre–post intervention study. Based on pilot observations and conservative estimates from previous exercise-intervention studies reporting changes in body-composition and lipid-profile outcomes (Milanović et al., 2015), the effect size was set at f = 0.30. According to Cohen’s conventional benchmarks, f = 0.25 represents a medium effect and f = 0.40 represents a large effect; therefore, f = 0.30 was considered a moderate-to-large expected effect for the group × time interaction. The input parameters were as follows: effect size *f* = 0.30, α = 0.05, statistical power = 0.85, number of groups = 4, number of measurements = 2, correlation among repeated measures = 0.50, and nonsphericity correction ϵ = 1.00. A total sample size of 40 participants was determined. This sample-size calculation was not linked to a single predefined primary outcome; rather, it was intended to provide basic power to detect a moderate group × time interaction across the selected cardiometabolic outcomes. Therefore, it should not be interpreted as providing adequate power for all secondary between-group comparisons across multiple cardiometabolic outcomes.

Forty sedentary male university students aged 18–28 years were recruited. Eligibility criteria included stable body weight during the preceding three months, no regular exercise habits, and no contraindications to exercise based on a general medical examination. Participants were excluded if they had a history of hereditary, cardiovascular, hepatic, renal, or pulmonary disease or were using weight-loss medication. Before training, all participants received a detailed explanation of the study procedures and provided written informed consent.

### 2.2 Study design

This study was a randomized, assessor-blinded controlled trial conducted at the Physical Performance Training Centre of Shenyang Sport University. Participants were allocated in a 1:1:1:1 ratio using a computer-generated random number sequence. After completing baseline testing, the 40 participants were randomly assigned to one of four groups: MISI, MILI, HISI, and HILI (n = 10 per group). During the 8-week intervention, all participants were instructed to maintain their usual dietary habits and daily lifestyle and to avoid additional exercise, weight-loss programs, or dietary interventions, in order to reduce the potential confounding effects of lifestyle changes. This design was intended to compare the relative effects of four active interval-training protocols rather than to estimate the overall effect of exercise training compared with no exercise. However, dietary intake and non-exercise physical activity were not objectively monitored using dietary records, accelerometers, pedometers, or activity logs.

Given that training intensity and interval duration are perceptible, blinding of participants and intervention administrators was not feasible. To minimize the risk of bias, outcome assessors and data analysts were kept blinded under de-identified conditions. The intervention and assessment teams were independent from each other, and all testing and training procedures were conducted in accordance with standardized operating protocols. Raw data were stored in anonymized coded form, and statistical analyses were performed using blinded group codes after database lock.

### 2.3 Intervention protocol

#### 2.3.1 Training equipment and intensity monitoring

All training sessions were performed on Keiser magnetic-resistance stationary bicycles (USA). Heart rate was recorded in real time using a Polar heart rate monitoring system, and exercise intensity was controlled according to target heart rate zones. HRmax was calculated using the following formula:


HRmax=208−0.7×age

Training intensity was classified as low, moderate, or high based on target heart rate zones. Participants were required to remain in the exercise area throughout each training session unless necessary. Rest and recovery phases consisted primarily of quiet sitting to avoid interference from extraneous physical activity on training load.

#### 2.3.2 Training schedule

Before the formal intervention, all participants completed a 2-week low-intensity adaptation phase to become familiar with the training equipment and procedures and to progressively establish exercise tolerance. Baseline outcome measurements were conducted after the adaptation phase and before the formal 8-week intervention. The formal intervention lasted 8 weeks, with three training sessions per week on Monday, Wednesday, and Friday. Each session included a 10-min warm-up, the assigned interval-training protocol, and a 10-min cool-down with soft tissue mobilization.

#### 2.3.3 Exercise protocol

Training intensity during the adaptation phase was set at 50% HRmax. During the formal intervention, the moderate-intensity groups trained at 60%–70% HRmax and the high-intensity groups at 85%–95% HRmax. During each session, heart rate was monitored in real time using a Polar heart rate monitoring system, and the resistance of the cycle ergometer was adjusted to help participants reach the prescribed target heart-rate zone. Training attendance and session completion were continuously recorded throughout the study.

In this study, the prescribed active-work load was approximately matched based on exercise prescription parameters to improve comparability when examining the relative effects of different combinations of exercise intensity and interval structure. Prescribed Active-Work Load was calculated using the following formula: (Garber et al., 2011).


Prescribed Active−Work Load=Exercise Intensity×Work Duration×Number of Sets.

In this formula, exercise intensity refers to the prescribed percentage of HRmax, and work duration refers only to the duration of active exercise bouts, excluding rest intervals and total session duration. Because exercise intensity was prescribed as a target heart-rate range, the prescribed active-work load was also expressed as a range. For example, the prescribed active-work load of the MISI protocol was calculated as 0.60–0.70 × 60 s × 7 sets = 252–294. It should be noted that this prescription-based active-work-load matching does not represent equivalence in complete physiological load, because recovery duration, total session duration, heart-rate kinetics, perceived exertion, and individual tolerance may differ across protocols (**Table 1**).

**Table 1:** Dose parameters of each intervention protocol.

Group	Intensity	Work duration	Rest duration	Interval total	Sets	Main interval duration	Total work duration	Prescribed active-work load
MISI	60%–70% HRmax	60 s	30 s	90 s	7 sets	630 s	420 s	252–294
MILI	60%–70% HRmax	60 s	120 s	180 s	7 sets	1260 s	420 s	252–294
HISI	85%–95% HRmax	30 s	30 s	60 s	10 sets	600 s	300 s	255–285
HILI	85%–95% HRmax	30 s	120 s	150 s	10 sets	1500 s	300 s	255–285

MISI, moderate-intensity short-interval training; MILI, moderate-intensity long-interval training; HISI, high-intensity short-interval training; HILI, high-intensity long-interval training; HRmax, maximal heart rate. The same group abbreviations apply in subsequent tables. The prescribed active-work load was calculated based on exercise prescription parameters and does not represent complete physiological load.

### 2.4 Outcome measures

#### 2.4.1 Body composition

All outcome measures were assessed at baseline and after the 8-week intervention. Participants were asked to abstain from alcohol the evening before testing and to maintain a fasting state of at least 2–4 hours prior to measurement. Body composition was assessed using a bioelectrical impedance body composition analyzer (InBody 760, InBody Co., Ltd., Seoul, Republic of Korea), including body weight, BFP, SMM, VFA, and BMR. All measurements were conducted during the same time of day to minimize the influence of circadian rhythms on results.

#### 2.4.2 Blood biochemical analysis

Serum lipid indicators were measured using commercial enzymatic assay kits according to the manufacturer’s instructions. The kits for TC/T-CHO (Cat. No. A111-1), TG (Cat. No. A110-1), HDL-C (Cat. No. A112-1), and LDL-C (Cat. No. A113-1) were all purchased from Nanjing Jiancheng Bioengineering Institute, Nanjing, China. Absorbance was measured using a microplate reader (Cmax Plus, Molecular Devices, USA). All assays were performed using the same batch of reagents under standard quality-control procedures.

### 2.5 Statistical analysis

All statistical analyses were performed using SPSS 26.0. Continuous variables were first assessed for normality using the Shapiro–Wilk test. Normally distributed data are presented as mean ± standard deviation ( $\bar{x}\pm s$); non-normally distributed data are expressed as median (interquartile range).

Two-way repeated-measures ANOVA (group × time) was used to evaluate differences between intervention conditions across pre- and post-intervention time points. The between-subject factor was group, including four levels: MISI, MILI, HISI, and HILI. The within-subject factor was time, including two levels: pre-intervention and post-intervention. Because the within-subject factor included only two time points, the assumption of sphericity was automatically satisfied.

Therefore, Mauchly’s test of sphericity and Greenhouse–Geisser correction were not applicable. When significant main effects or interaction effects were identified, *post-hoc* comparisons were performed with Bonferroni correction. Effect sizes were reported as partial eta squared ( $\eta_{p}^{2}$). All statistical tests were two-tailed, with the significance level set at α = 0.05.

## 3 Results

### 3.1 Intervention adherence and adverse events

All 40 participants completed the 8-week intervention, with no dropouts. Each participant completed all 24 prescribed training sessions; therefore, attendance and session adherence were 100% in all four groups. No training-related adverse events were observed during the intervention. The percentage of time spent within the target heart-rate zone was not available because the original heart-rate data were not systematically exported for this purpose. Therefore, although heart rate was monitored in real time during training and cycling resistance was adjusted to help participants reach the prescribed zones, full adherence to the target heart-rate zones could not be retrospectively verified.

### 3.2 Morphological and energy metabolism indices

#### 3.2.1 BFP

Data from all groups conformed to a normal distribution. Two-way repeated-measures ANOVA revealed a significant main effect of time (*F* = 134.697, *p* < 0.01, η_p_² = 0.789) and a significant group × time interaction effect (*F* = 9.586, *p* < 0.01, η_p_² = 0.444) on body fat percentage, while the main effect of group was not significant (*F* = 1.092, *p* = 0.365, η_p_² = 0.083). Simple effects analysis indicated that BFP decreased significantly in all groups following the intervention (all *p* < 0.01), with no significant between-group differences (**Table 2**).

**Table 2:** Changes in BFP across groups ( $\bar{x}\pm SD$, %).

Group	Pre-intervention	Post-intervention	Change from baseline	95% CI for change
MISI	21.32 ± 3.10	19.93 ± 2.81^**^	-1.388	[-2.108, -0.668]
MILI	23.14 ± 4.23	22.13 ± 4.05^**^	-1.008	[-1.728, -0.288]
HISI	25.08 ± 3.88	21.62 ± 3.55^**^	-3.460	[-4.180, -2.740]
HILI	23.00 ± 3.90	20.62 ± 2.86^**^	-2.380	[-3.100, -1.660]

* and ** indicate p < 0.05 and p < 0.01 versus pre-intervention within the same group, respectively.

#### 3.2.2 VFA

VFA showed a significant main effect of time (*F* = 259.31, *p* < 0.01, η_p_² = 0.878) and a significant interaction effect (*F* = 42.541, *p* < 0.01, η_p_² = 0.780), while the main effect of group was not significant (*F* = 2.521, *p* = 0.073, η_p_² = 0.174). All groups showed significant reductions following the intervention (*p* < 0.01), and *post-hoc* analysis showed that post-intervention VFA was significantly higher in MILI than in HISI (*p* = 0.030) (**Table 3**).

**Table 3:** Changes in VFA across groups ( $\bar{x}\pm SD$, cm²).

Group	Pre-intervention	Post-intervention	Change from baseline	95% CI for change
MISI	32.53 ± 5.64	30.99 ± 5.70^**^	-1.540	[-2.394, -0.686]
MILI	36.16 ± 6.84	35.15 ± 7.01^**#^	-1.010	[-1.864, -0.156]
HISI	31.25 ± 6.21	24.23 ± 4.82^**^	-7.020	[-7.874, -6.161]
HILI	35.47 ± 8.65	31.48 ± 7.91^**^	-3.990	[-4.848, -3.136]

* and ** indicate p < 0.05 and p < 0.01 versus pre-intervention within the same group, respectively; # and ## indicate p < 0.05 and p < 0.01 versus HISI at post-intervention, respectively.

#### 3.2.3 SMM

SMM showed a significant main effect of time (*F* = 34.299, *p* < 0.01, η_p_² = 0.488), but neither the interaction effect (*F* = 0.051, *p* = 0.985, η_p_² = 0.004) nor the main effect of group (*F* = 1.194, *p* = 0.326, η_p_² = 0.090) was significant. Skeletal muscle mass increased significantly in all groups following the intervention (*p* < 0.05), with no significant between-group differences (**Table 4**).

**Table 4:** Changes in SMM across groups ( $\bar{x}\pm SD$, kg).

Group	Pre-intervention	Post-intervention	Change from baseline	95% CI for change
MISI	35.51 ± 3.73	36.83 ± 3.45^**^	1.315	[0.347, 2.283]
MILI	35.55 ± 3.09	36.85 ± 3.14^*^	1.296	[0.328, 2.264]
HISI	33.02 ± 4.35	34.52 ± 4.26^**^	1.501	[0.553, 2.469]
HILI	33.96 ± 2.82	35.44 ± 2.97^**^	1.480	[0.512, 2.448]

* and ** indicate p < 0.05 and p < 0.01 versus pre-intervention within the same group, respectively.

#### 3.2.4 BMR

BMR showed a significant main effect of time (*F* = 31.736, *p* < 0.01, η_p_² = 0.469), while the group × time interaction (*F* = 1.692, *p* = 0.186, η_p_² = 0.124) and the main effect of group (*F* = 0.604, *p* = 0.617, η_p_² = 0.048) were not significant. BMR increased significantly in MILI, HISI, and HILI following the intervention, whereas the increase in MISI did not reach statistical significance (**Table 5**).

**Table 5:** Changes in BMR across groups ( $\bar{x}\pm SD$, kcal/day).

Group	Pre-intervention	Post-intervention	Change from baseline	95% CI for change
MISI	1657.60 ± 144.73	1665.80 ± 145.63	8.200	[-5.948, 22.348]
MILI	1680.70 ± 145.14	1697.60 ± 146.53^**^	16.900	[2.752, 31.048]
HISI	1647.80 ± 205.14	1694.20 ± 210.86^**^	24.800	[10.652, 39.948]
HILI	1592.40 ± 105.35	1621.10 ± 107.10^**^	28.700	[14.552, 42.848]

* and ** indicate p < 0.05 and p < 0.01 versus pre-intervention within the same group, respectively.

### 3.3 Lipid profile and lipid metabolism indices

#### 3.3.1 TC

TC showed a significant main effect of time (*F* = 170.848, *p* < 0.01, η_p_² = 0.826) and a significant interaction effect (*F* = 6.658, *p* = 0.001, η_p_² = 0.357), while the main effect of group was not significant (*F* = 0.447, *p* = 0.721, η_p_² = 0.036). TC levels decreased significantly in all groups following the intervention (*p* < 0.01), with no significant between-group differences (**Table 6**).

**Table 6:** Changes in TC across groups ( $\bar{x}\pm SD$, mmol/L).

Group	Pre-intervention	Post-intervention	Change from baseline	95% CI for change
MISI	4.42 ± 0.60	4.25 ± 0.60^**^	-0.164	[-0.243, -0.086]
MILI	4.61 ± 0.78	4.44 ± 0.77^**^	-0.169	[-0.247, -0.090]
HISI	4.78 ± 0.30	4.46 ± 0.36^**^	-0.326	[-0.404, -0.248]
HILI	4.63 ± 0.48	4.28 ± 0.45^**^	-0.350	[-0.429, -0.272]

* and ** indicate p < 0.05 and p < 0.01 versus pre-intervention within the same group, respectively.

#### 3.3.2 TG

TG showed a significant main effect of time (*F* = 312.961, *p* < 0.01, η_p_² = 0.897) and a significant interaction effect (*F* = 59.461, *p* < 0.01, η_p_² = 0.832), while the main effect of group was not significant (*F* = 1.104, *p* = 0.360, η_p_² = 0.084). TG decreased significantly in all groups following the intervention (*p* < 0.01). *Post-hoc* analysis showed that post-intervention TG was significantly higher in MISI than in HISI (*p* = 0.049) (**Table 7**).

**Table 7:** Changes in TG across groups ( $\bar{x}\pm SD$, mmol/L).

Group	Pre-intervention	Post-intervention	Change from baseline	95% CI for change
MISI	1.50 ± 0.15	1.42 ± 0.15^**#^	-0.076	[-0.125, -0.027]
MILI	1.35 ± 0.28	1.25 ± 0.32^**^	-0.100	[-0.149, -0.051]
HISI	1.53 ± 0.33	1.04 ± 0.27^**^	-0.483	[-0.532, -0.434]
HILI	1.43 ± 0.17	1.22 ± 0.14^**^	-0.198	[-0.247, -0.149]

* and ** indicate p < 0.05 and p < 0.01 versus pre-intervention within the same group, respectively; # and ## indicate p < 0.05 and p < 0.01 versus HISI at post-intervention, respectively.

#### 3.3.3 LDL-C

LDL-C showed a significant main effect of time (*F* = 49.812, *p* < 0.001, η_p_² = 0.580) and a significant interaction effect (*F* = 5.832, *p* = 0.002, η_p_² = 0.327), while the main effect of group was not significant (*F* = 1.138, *p* = 0.347, η_p_² = 0.087). Simple effects analysis indicated that LDL-C decreased significantly only in HISI and HILI following the intervention (*p* < 0.01), with no significant changes observed in MISI and MILI (**Table 8**).

**Table 8:** Changes in LDL-C across groups ( $\bar{x}\pm SD$, mmol/L).

Group	Pre-intervention	Post-intervention	Change from baseline	95% CI for change
MISI	2.25 ± 0.40	2.20 ± 0.37	-0.061	[-0.129, 0.007]
MILI	2.58 ± 0.32	2.54 ± 0.37	-0.037	[-0.105, 0.031]
HISI	2.44 ± 0.35	2.24 ± 0.47^**^	-0.196	[-0.264, -0.128]
HILI	2.48 ± 0.48	2.31 ± 0.50^**^	-0.179	[-0.247, -0.111]

* and ** indicate p < 0.05 and p < 0.01 versus pre-intervention within the same group, respectively.

#### 3.3.4 HDL-C

HDL-C showed significant main effects of time (*F* = 127.733, *p* < 0.01, η_p_² = 0.780) and group (*F* = 3.633, *p* = 0.022, η_p_² = 0.232), as well as a significant interaction effect (*F* = 4.296, *p* = 0.011, η_p_² = 0.264). HDL-C increased significantly in all groups following the intervention (*p* < 0.05). At post-intervention, HDL-C levels in HISI and HILI were significantly higher than those in MILI (*p* = 0.012 and *p* = 0.018, respectively) (**Table 9**).

**Table 9:** Changes in HDL-C across groups ( $\bar{x}\pm SD$, mmol/L).

Group	Pre-intervention	Post-intervention	Change from baseline	95% CI for change
MISI	1.16 ± 0.10	1.34 ± 0.13^**^	0.182	[0.126, 0.237]
MILI	1.18 ± 0.10	1.25 ± 0.07^*^	0.069	[0.014, 0.125]
HISI	1.26 ± 0.17	1.45 ± 0.13^**†^	0.183	[0.128, 0.239]
HILI	1.25 ± 0.09	1.44 ± 0.15^**†^	0.184	[0.128, 0.239]

* and ** indicate p < 0.05 and p < 0.01 versus pre-intervention within the same group, respectively; † and †† indicate p < 0.05 and p < 0.01 versus MILI at post-intervention, respectively.

## 4 Discussion

### 4.1 Main findings

This randomized controlled trial compared the effects of four interval-training protocols combining different exercise intensities, moderate versus high intensity, and interval structures, short versus long intervals, on body composition, energy metabolism, and lipid-profile-related cardiometabolic indicators in sedentary young males. After the 8-week intervention, BFP, VFA, TC, and TG decreased significantly in all groups, whereas SMM and HDL-C increased significantly. These findings are consistent with previous studies reporting that regular interval training is associated with favorable changes in early cardiometabolic risk-related indicators in sedentary young males (Musa et al., 2009; Sun et al., 2024).

*Post-hoc* analyses showed that post-intervention VFA was significantly lower in the HISI protocol than in the MILI protocol, and TG was significantly lower in the HISI protocol than in the MISI protocol. In addition, HDL-C levels were significantly higher in the HISI and HILI protocols than in the MILI protocol, whereas LDL-C decreased significantly only in the two high-intensity protocols. Taken together, these findings suggest that the four active interval-training protocols were associated with favorable changes in several cardiometabolic indicators in sedentary young males, while exercise intensity and interval structure may influence specific metabolic responses differently.

### 4.2 Effects of interval training on BFP and VFA

The present study found that all four 8-week interval-training protocols significantly reduced BFP in sedentary young males. This finding is broadly consistent with previous evidence suggesting that regular interval training may increase total energy expenditure, enhance skeletal muscle metabolic demand, promote substrate mobilization, and further improve adipose tissue function (Maillard et al., 2018), thereby optimizing body composition (Westerterp, 2018; Kolnes et al., 2021). This response may be related to the long-term insufficient physical activity, low daily energy expenditure, and limited lipid turnover capacity commonly observed in sedentary individuals (Tremblay et al., 2010).

Compared with BFP, changes in VFA showed more pronounced differences across protocols. Although all four interval-training protocols significantly reduced VFA, the HISI protocol showed the largest reduction, and post-intervention VFA was significantly lower in the HISI protocol than in the MILI protocol. These findings suggest that exercise intensity may be an important factor associated with VFA reduction, while interval structure may further modulate the magnitude of metabolic stimulation. Previous studies have suggested that a threshold effect may exist between exercise intensity and visceral fat reduction, whereby higher-intensity exercise may increase post-exercise energy expenditure and fat oxidation (Panissa et al., 2021; Jiang et al., 2024). In addition, shorter recovery intervals may expose the body to sustained cardiovascular and metabolic demands before full recovery is achieved (Maillard et al., 2018). During this process, the body repeatedly undergoes cycles of heart-rate elevation, metabolic stress accumulation, and recovery regulation within a relatively short period, which may increase overall metabolic perturbation. Such repeated metabolic stimulation may further enhance post-exercise energy expenditure, promote lipid mobilization and substrate turnover, and ultimately contribute to VFA reduction (Atakan et al., 2022).

### 4.3 Effects of interval training on SMM and BMR

The present study found that all four interval-training protocols increased SMM, with relatively consistent improvements across groups. This finding is consistent with previous studies reporting limited differences between HIIT and moderate-intensity continuous training in improving lean mass (Guo et al., 2023; Khodadadi et al., 2023). This phenomenon may be related to the “initial adaptation effect” that occurs when sedentary individuals transition from a low-activity state to regular exercise. During this process, even when training protocols differ in intensity and structure, the body may first exhibit relatively consistent basic adaptations once regular exercise is established (Hughes et al., 2018). These adaptations may include increased muscle contraction frequency, enhanced motor-unit recruitment, greater local metabolic demand, and improved skeletal muscle metabolic capacity (Caparrós-Manosalva et al., 2023). The 8-week intervention period may not have been sufficient for protocol-specific muscular adaptations to become clearly differentiated; therefore, no significant between-group differences were observed in the magnitude of SMM improvement.

For BMR, the MILI, HISI, and HILI protocols showed significant post-intervention increases, whereas the MISI protocol showed an upward trend that did not reach statistical significance. Regular interval training may influence BMR by increasing muscle activity, elevating skeletal muscle energy demand, enhancing oxidative metabolic capacity, and promoting adaptations related to fat-free mass (Mackenzie-Shalders et al., 2020). As a training modality characterized by relatively high metabolic stimulation, HIIT may affect energy-metabolism-related indicators by improving cardiorespiratory fitness, enhancing muscular oxidative capacity, and optimizing body composition (Amaro-Gahete et al., 2020). It should be noted that BMR in this study was estimated using a body composition analyzer rather than directly measured by indirect calorimetry. Therefore, these changes should not be directly interpreted as physiological increases in true resting metabolic rate.

### 4.4 Effects of interval training on lipid profile

The present findings suggest that exercise intensity and interval structure may differentially influence changes in lipoprotein components (Wood et al., 2019). Previous evidence has shown that exercise training can improve lipid-profile indicators, including TC, HDL-C, LDL-C, TG, and VLDL, although the overall magnitude of these improvements is generally moderate or small (Smart et al., 2025).

In the present study, TC decreased significantly after the intervention in all four protocols, with no significant between-group differences. This suggests that the different interval-training protocols may have comparable effects on TC reduction. As a comprehensive indicator reflecting total plasma cholesterol, TC is usually influenced by multiple factors, including intervention duration, total energy expenditure, baseline lipid status, and changes in body fat (Katzmarzyk et al., 2001; Smart et al., 2025).Therefore, its improvement is difficult to attribute to a single training variable.

For TG, all four protocols showed significant post-intervention reductions, and TG was significantly lower in the HISI protocol than in the MISI protocol. This suggests that the HISI protocol may induce a more favorable training response for TG reduction. One possible explanation is that high-intensity exercise may substantially increase acute energy demand and skeletal muscle metabolic load, while shorter recovery intervals may require participants to enter the next exercise bout before full recovery is achieved (Lira et al., 2012).This pattern may repeatedly induce heart-rate elevation, increased oxygen demand, and accumulated metabolic stress within a single session. Under such repeated metabolic perturbation, post-exercise TG metabolic efficiency may be further enhanced (Sun et al., 2024).In addition, regular exercise may promote TG reduction by increasing skeletal muscle lipoprotein lipase activity (Matsumoto et al., 2019),enhancing post-exercise TG clearance (Loh et al., 2020),and reducing hepatic VLDL-TG secretion (Plaisance and Fisher, 2014).

For LDL-C, significant post-intervention reductions were observed only in the HISI and HILI protocols, whereas no significant changes were found in the MISI or MILI protocols. This finding is consistent with previous evidence and suggests that LDL-C may be more sensitive to exercise stimulus intensity (Mc C C et al., 2023).Previous studies have indicated that high-intensity training may indirectly influence LDL-C levels by improving insulin sensitivity (Ryan et al., 2020),promoting fat reduction, reducing hepatic lipid burden, and enhancing lipoprotein metabolic regulation (Tsekouras et al., 2008; Smart et al., 2026).

For HDL-C, all four protocols showed significant post-intervention increases, and HDL-C levels were significantly higher in the two high-intensity protocols than in the MILI protocol. This suggests that higher exercise intensity may further enhance the HDL-C response. The regulation of HDL-C involves multiple processes (Franczyk et al., 2023), including lipoprotein lipase activity, apolipoprotein A1 synthesis, ABCA1 transporter expression, and reverse cholesterol transport (Marques et al., 2018; Sucharski and Koenig, 2022). Therefore, the present study only indicates favorable changes in HDL-C levels and does not allow further inference regarding improvements in HDL function.

### 4.5 Strengths and limitations

This study has several strengths. First, it used a four-arm randomized controlled design to compare the combined effects of exercise intensity, moderate versus high intensity, and interval structure, short versus long intervals, rather than focusing only on a single HIIT protocol versus conventional training. Second, body composition, BMR, and lipid profile indicators were included, allowing cardiometabolic risk-related changes to be evaluated from multiple perspectives. Third, training frequency, intervention duration, and HRmax-based intensity control were kept consistent across groups, which improved the comparability of the four protocols.

Several limitations should also be acknowledged. First, the sample size was relatively small, with only 10 participants in each group, and all participants were sedentary young males. This may limit the statistical power, the stability of between-group findings, and the generalizability of the results. Therefore, the significant between-group differences should be interpreted as preliminary. Second, the study did not include a non-exercise control group, and dietary intake and daily physical activity were not objectively monitored, which may have introduced potential confounding effects. Third, energy metabolism and related mechanistic indicators were not directly measured, limiting further interpretation of the underlying mechanisms. Moreover, although real-time heart-rate monitoring was used during each training session to guide exercise intensity, the raw heart-rate data were not systematically exported or archived. As a result, we were unable to quantify the proportion of training time spent within the prescribed target heart-rate zones or fully verify participants’ adherence to the intended intensity ranges. This limitation should be considered when interpreting potential differences among protocols with different prescribed exercise intensities. Fourth, prescription-parameter-based active-work-load matching was used in this study rather than direct physiological-load matching. Therefore, differences in recovery duration, total session duration, heart-rate responses, perceived exertion, internal load, and individual tolerance may still have existed across protocols.

## 5 Conclusion

Eight weeks of interval training was associated with favorable changes in selected body composition and lipid-profile indicators in sedentary young males. Compared with the moderate-intensity protocols, the high-intensity protocols showed more favorable changes in selected outcomes, particularly VFA, TG, and LDL-C. A shorter interval structure may further contribute to favorable metabolic responses in some indicators; however, these between-group findings should be interpreted cautiously because all groups received active exercise interventions and no non-exercise control group was included. From a practical perspective, the HISI protocol may represent a promising option for improving selected cardiometabolic risk indicators related to visceral adiposity and lipid metabolism in sedentary young males. However, its applicability may be influenced by individual baseline fitness and tolerance to high-intensity exercise. For individuals with limited exercise experience or low tolerance to high-intensity training, moderate-intensity interval training may remain a safe, feasible, and effective initial strategy.

## Data Availability

The original contributions presented in the study are included in the article/supplementary material. Further inquiries can be directed to the corresponding author.
